# Altered White Matter and Layer VIb Neurons in Heterozygous *Disc1* Mutant, a Mouse Model of Schizophrenia

**DOI:** 10.3389/fnana.2020.605029

**Published:** 2020-12-15

**Authors:** Shin-Hwa Tsai, Chih-Yu Tsao, Li-Jen Lee

**Affiliations:** ^1^School of Medicine, National Taiwan University, Taipei, Taiwan; ^2^Graduate Institute of Anatomy and Cell Biology, National Taiwan University, Taipei, Taiwan; ^3^Institute of Brain and Mind Sciences, National Taiwan University, Taipei, Taiwan; ^4^Neurobiology and Cognitive Science Center, National Taiwan University, Taipei, Taiwan

**Keywords:** animal model, layer VIb, CTGF, subplate neuron, white matter neuron, neuron morphology

## Abstract

Increased white matter neuron density has been associated with neuropsychiatric disorders including schizophrenia. However, the pathogenic features of these neurons are still largely unknown. Subplate neurons, the earliest generated neurons in the developing cortex have also been associated with schizophrenia and autism. The link between these neurons and mental disorders is also not well established. Since cortical layer VIb neurons are believed to be the remnant of subplate neurons in the adult rodent brain, in this study, we aimed to examine the cytoarchitecture of neurons in cortical layer VIb and the underlying white matter in heterozygous *Disc1* mutant (Het) mice, a mouse model of schizophrenia. In the white matter, the number of NeuN-positive neurons was quite low in the external capsule; however, the density of these cells was found increased (54%) in Het mice compared with wildtype (WT) littermates. The density of PV-positive neurons was unchanged in the mutants. In the cortical layer VIb, the density of CTGF-positive neurons increased (21.5%) in Het mice, whereas the number of Cplx3-positive cells reduced (16.1%) in these mutants, compared with WT mice. Layer VIb neurons can be classified by their morphological characters. The morphology of Type I pyramidal neurons was comparable between genotypes while the dendritic length and complexity of Type II multipolar neurons were significantly reduced in Het mice. White matter neurons and layer VIb neurons receive synaptic inputs and modulate the process of sensory information and sleep/arousal pattern. Aberrances of these neurons in *Disc1* mutants implies altered brain functions in these mice.

## Introduction

Schizophrenia is a major chronic mental disorder that occurs in about 1% of the general population (McGrath et al., 2004). It is diagnosed by clinical signs and syndromes, including hallucinations, delusions, disorganized thought and speech, flattening effect, social withdrawal alogia, and deficits in cognitive functions. While the pathogenic mechanisms of schizophrenia are still not fully understood, both genetic and environmental factors are thought to play important roles in triggering the disease (van Os et al., 2010).

*Disrupted-In-Schizophrenia 1* (*DISC1*) is one of the susceptible genes of schizophrenia. A defective variant of *the DISC1* gene first discovered from a Scottish pedigree, and carried a balanced translocation (1;11; q42.1; q14.3) that was strongly associated with major mental illness such as schizophrenia, depression, and bipolar disorder (St Clair et al., 1990; Millar et al., 2000). The linkage between *DISC1* gene abnormalities and schizophrenia has also been found in Taiwanese families (Hwu et al., 2003; Liu et al., 2009). DISC1 proteins are expressed in the cerebral cortex, hippocampus, hypothalamus, cerebellum, and brainstem in humans (Kirkpatrick et al., 2006); while in rodents, the expression of Disc1 has also been explored. Disc1 protein is detected in the cerebrum, cerebellum, brainstem, and various internal organs of mice as early as embryonic day 13. In the cerebrum, the expression of Disc1 increases during the perinatal period with a peak during the first postnatal week and gradually declines thereafter (Kuroda et al., 2011). The expression profile suggests a role of Disc1 in brain development. In addition to neurons, DISC1 is also expressed in astrocytes, oligodendrocytes, and microglia (Seshadri et al., 2010). DISC1 protein contains the N-terminal globular head domain and the coiled-helical coil-containing C-terminal tail domain. It has been demonstrated to interact with a great number of proteins; therefore, it is suggested to be a hub for multiple signaling pathways (Brandon and Sawa, 2011). DISC1 and its binding partners have been shown to regulate several cellular functions, such as neuronal migration, axon extension, dendritic differentiation, mitochondria motility, cargo transport, and synaptic plasticity. A variety of psychiatric phenotypes may be derived if DISC1 protein is disrupted (Brandon and Sawa, 2011; Bradshaw and Porteous, 2012; Lipina and Roder, 2014; Shao et al., 2017; Tropea et al., 2018).

Schizophrenia is a human-specific mental disorder; however, animal models could be used to replicate some aspects of the disease, especially the impact of certain susceptibility genes (Dahoun et al., 2017). Several mouse models have been made to mimic the disruption in *the DISC1* gene (Jaaro-Peled, 2009; Tomoda et al., 2016). We had established a *Disc1* mutant mouse model, in which a 25-bp deletion variant of 129S6/SvEv *Disc1* gene was brought to the C57BL/6J background by generations of backcrossing (Juan et al., 2014). Compared with other *Disc1* mutant mice, our model exhibits relatively moderate abnormalities, suggesting that these mice are in a prodromal status (Juan et al., 2014; Baskaran et al., 2020). This model is therefore suitable for studies concerning the impact of environmental risk factors. It has been suggested that structural abnormalities in the brain during neurodevelopmental mainly attributed to a gene mutation such as *DISC1* deficiency may pave a way for functional impairments elicited by later environmental insults that promote the manifestation of psychotic symptoms (Narayan et al., 2013).

The cortical subplate zone is located between the developing cortical plate and the axons of afferent nerves (Allendoerfer and Shatz, 1994) and contains cells that are among the earliest generated neurons in the developing cortex and play important roles in cortical development (Kostovic and Rakic, 1980; Chun and Shatz, 1989; Bayer and Altman, 1990; McQuillen and Ferriero, 2005; Bystron et al., 2008; Judaš et al., 2010; Kanold and Luhmann, 2010; Hoerder-Suabedissen and Molnár, 2013; Ohtaka-Maruyama et al., 2018; Ohtaka-Maruyama, 2020). For example, these neurons set up the first connection between the thalamus and cortex that play crucial roles in the maturation of the inhibitory circuit and the organization of the neocortex (Kanold and Shatz, 2006; Kanold and Luhmann, 2010; Hoerder-Suabedissen and Molnár, 2015). Many subplate neurons go through programmed cell death after birth. The number of subplate neurons declines and remains as white matter and layer VIb neurons (Chun and Shatz, 1989; García-Marín et al., 2010; Judaš et al., 2010; Marx et al., 2017; Hoerder-Suabedissen et al., 2018). A genetic study showed the expression profile of mouse subplate neurons associated with autism and schizophrenia (Hoerder-Suabedissen et al., 2013). Abnormalities in subplate or white matter neurons have been linked to mental disorders such as schizophrenia (Duchatel et al., 2016, 2019; Serati et al., 2019; Kubo, 2020); however, the pathogenic role of remnant subplate neurons is still largely unknown (Judaš et al., 2010).

In this study, we examined the cytoarchitecture of neurons in cortical layer VIb and the underlying white matter in heterozygous *Disc1* mutant (Het) mice. If the abnormalities of these neurons found in this genetic mouse model of schizophrenia could replicate the findings in patients with schizophrenia (Duchatel et al., 2019; Serati et al., 2019; Kubo, 2020), then we may have a promising rodent model for investigating molecular and cellular events which might contribute to the pathogenesis of schizophrenia. Our results showed alterations in the density of layer VIb and white matter neurons as well as the morphology of layer VIb neurons in *Disc1* mutant mice and suggested a pathogenic feature of neuropsychiatric disorders.

## Materials and Methods

### Animals

Mice were housed in the Laboratory Animal Center of the College of Medicine, National Taiwan University (NTU) approved by AAALAC, under a 12:12 light-dark cycle with free access to food and water. Young adult (10–12 weeks old) male wild-type (WT) and heterozygous *Disc1* (Het) mice were used in this study. Mice were genotyped using genomic DNA isolated from the toes and PCR-mediated methods as previously described (Juan et al., 2014). Animal experiments were performed following the guideline of the Institutional Animal Care and Use Committee of the College of Medicine, NTU. Efforts were constantly made to minimize animal discomfort and the number of mice used.

### Histological and Morphological Examinations

WT and Het male mice were anesthetized and transcardially perfused with 0.1 M phosphate-buffered saline (PBS; pH 7.4) followed by 4% paraformaldehyde in 0.1 M PBS. Whole brains were removed and post-fixed in the same fixation overnight. Then, the brains were stored in 0.1% NaN_3_ in 0.1 M PBS.

### Immunohistochemistry

Brain samples were sectioned coronally at 30 μm with a vibratome (VT1000S, Leica Biosystems, Wetzlar, Germany) and one out of 12 sections which ranged from Bregma 1.00 mm to 0.00 mm was used for immunohistochemistry. The free-floating sections were first bleached in 0.1% H_2_O_2_ in 0.1 M PBS for 15 min to suppress endogenous peroxidase activity and washed in 0.1 M PBS. Then, the sections were incubated in the blocking solution containing 4% normal goat serum, 1% bovine serum albumin, and 0.4% Triton X-100 in 0.1 M PBS for 2 h to prevent non-specific binding. Afterward, the sections were incubated overnight with primary antibodies in diluted blocking solution on a shaker. The following primary antibodies were used: mouse monoclonal anti-NeuN (1:1,000, RRID: AB_177621; MAB377B; Millipore, Merck KGaA, Darmstadt, Germany), mouse polyclonal anti-Parvalbumin (PV; 1:3,000, RRID: AB_477329; P3088; Sigma–Aldrich, Merck KGaA), goat polyclonal anti-CTGF (1:1,000; RRID: AB_638805; sc-14939; Santa Cruz, CA, USA), mouse polyclonal anti-Nurr1 (1:250, RRID: AB_2153894; AF2156; R&D Systems, Minneapolis, MN, USA) and rabbit polyclonal anti-Cplx3 (1:2,000, RRID: AB_2281240, Cat# 122 302; Synaptic Systems, Göttingen, Germany). After washed with 0.1 M PBS, the sections were incubated in biotinylated secondary antibodies in 0.4% Triton X-100 for 1 h. The following secondary antibodies were used: biotinylated goat anti-mouse, biotinylated goat anti-rabbit, and biotinylated rabbit anti-goat (1:500, Jackson ImmunoResearch, West Grove, PA, USA). After washes, the sections were incubated with the reagents of the Vectastin (ABC kit, Vector Laboratories, Burlingame, CA, USA) for 1 h. Finally, sections were reacted with 2 mg/ml of 3′,3′-diaminobenzidine with 0.01% H_2_O_2_ in PBS, washed in PBS, and mounted.

### Measurement of Neurons in the Gray and White Matter

Images of immunostained coronal sections were taken from the somatosensory cortex. Counting squares of 100 × 100 μm were used to estimate the densities of NeuN- and PV-immunopositive signals in the upper, middle, and lower regions of the cortex, while a frame of 25 × 100 μm was used to count CTGF-, Cplx3-, and Nurr1-positive cells in the cortical layer VIb. To evaluate the density of cells in the white matter, the areas of cingulum (cg) and external capsule (ec) underneath the somatosensory cortex were measured according to a mouse brain atlas (Paxinos and Franklin, 2008). The junction between gray matter and white matter was determined based on the immunostainings of NeuN. The numbers of NeuN- and PV-positive neurons in the cg and ec were counted. To measure the distribution of cortical neurons, the thickness of the cortex was equally subdivided into 20 counting bins, starting from the pia surface to the border of white matter. Due to the different densities of immunopositive signals, the width of the counting bin was set 50 μm for NeuN-positive and 100 μm for PV-positive cells, respectively. The numbers of cells were totaled and the proportion of cells in each bin was calculated and represented as a percentage across all 20 counting bins. In this counting system, bins 1–2 roughly correspond to cortical layer I, bins 3–6 for layer II/III, bin 7–8 for layer IV, bins 9–14 for layer V, bins 15–19 for layer VIa, and bin 20 for layer VIb, respectively.

### Golgi-Cox Staining

Brain samples were collected and bathed in an impregnation solution (FD Rapid GolgiStain kit, NeuroTechnologies, Ellicott City, MD, USA) at room temperature for 3 weeks. Impregnated samples were transferred to ddH_2_O for at least 1 day and then serially sectioned at 150 μm using a vibratome (Leica). The sections were bathed in a mixture of developer and fixer solution from the same kit, then washed, and mounted. The images of subplate neurons in layer VIb of the somatosensory cortex were collected under a light microscope (Olympus, Tokyo, Japan) with a 20× objective lens for dendritic analysis. The stacks of images were obtained using the StereoInvestigator system (MicroBrightField Bioscience, Williston, VT, USA) and the morphology of subplate neurons was reconstructed with Neurolucida software (MicroBrightField). The Sholl and branched structure analyses provided in Neurolucida Explorer software were used to quantify the topological parameters such as the number of primary dendrites and branching nodes and size-related parameters, including soma size, convex hull volume, dendritic length, as well as intersections and nodes in relation to the distance from the soma.

### Data Analysis

Quantitative data of distribution, cell density, thickness, and morphometric parameters were measured blindly to the genotype of mice. The data were analyzed by two-tailed Student’s *t*-test and were presented as mean ± SEM. *p* < 0.05 was considered significance (*p < 0.05, **p < 0.01, ***p < 0.001). Statistics of the dendritic polar plot were done using R version 3.4.3 (RCoreTeam, 2017) and the ggplot2 (Wickham, 2016) package.

## Results

### Patterns of Cortical Neurons

**Figure 1:**
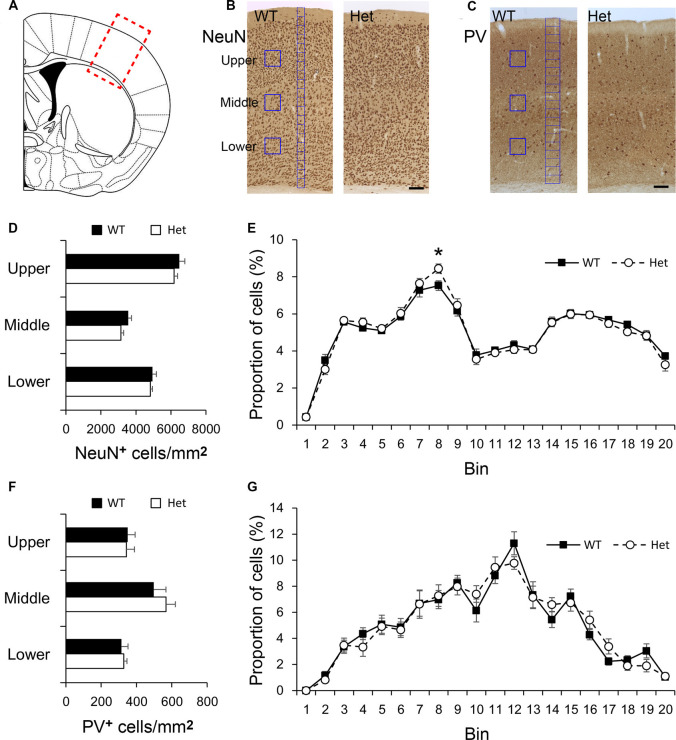
Density and distribution of neurons in the somatosensory cortex. The region of interest was labeled by a red rectangle in the somatosensory cortex **(A)**. Cortical neurons were immunostained with NeuN **(B)**, or Parvalbumin (PV, **C**). The densities of NeuN-positive cortical neurons in counting boxes (blue squares in **B**) in the upper, middle, and lower regions were measured **(D)**. The distribution of NeuN-positive cortical neurons was measured in 20 counting bins with the width of 50 μm (blue bins in **B**), the counted neurons in each bin were converted into the proportion of total cells **(E)**. The densities of PV-positive cortical neurons in the upper, middle, and lower regions were measured **(F)**. The distribution of PV-positive cortical neurons was measured using 20 counting bins with a width of 100 μm (blue bins in **C**) **(G)**. Scale bar = 100 μm in panel **(C)**. *N* = 8 wildtype (WT) and 7 Het mice. Results are means ± SEM. *p < 0.05.

Disc1 has been suggested to play a role in cortical neuron migration (Narayan et al., 2013; Muraki and Tanigaki, 2015). We first examined the density and distribution of cortical neurons in the somatosensory cortex (Figure 1A) of WT and *Disc1* Het mice. Neurons in the somatosensory cortex were immunostained with a pan-neuronal marker, NeuN (Figure 1B), and a marker for inhibitory interneurons, parvalbumin (PV, Figure 1C), and then quantified by counting of immunolabeled cells. The densities of NeuN-positive cells measured in upper, middle, and lower cortical regions were comparable between WT and *Disc1* Het mice (Figure 1D). To measure the distribution of cortical neurons, the thickness of the cortex was divided into 20 counting bins and the counted neurons were totaled and converted into the proportion of cells in each bin (Yu et al., 2019). We regarded bins 1–2 as the layer I of the cerebral cortex; bins 3–6 as layers II/III; bins 7–8 as layer IV; bins 9–14 as layer V, bins 15–19 as layer VIa and bin 20 as layer VIb (Figure 1B). The distribution of NeuN-positive neurons was similar between genotypes in most counting bins, except for a difference in bin 8, the layer IV, between WT and Het groups (Figure 1E). PV was used as a marker for inhibitory neurons (Figure 1C), which make up about 40% of the interneurons in the somatosensory cortex (Rudy et al., 2011). The densities of PV-positive cortical neurons in the upper, middle, and lower regions were comparable between WT and *Disc1* Het mice (Figure 1F). The distributions of PV-expressing neurons were also similar between groups (Figure 1G). These results indicated that the *Disc1* haploinsufficiency does not significantly affect the density of cortical neurons while only a subtle difference in the distribution of layer IV neurons was noted.

### The Density of White Matter Neurons

**Figure 2:**
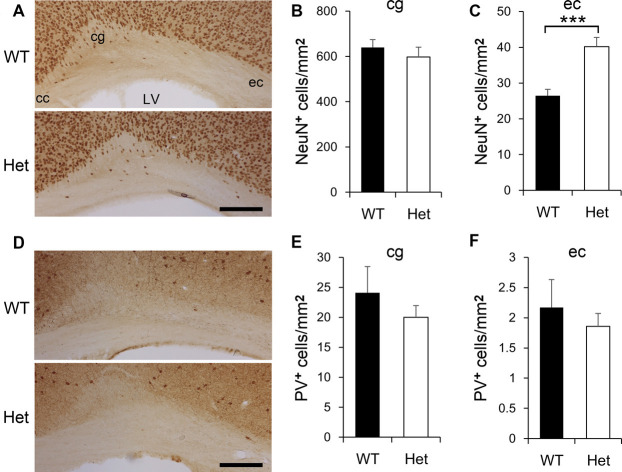
The density of white matter neurons in WT and *Disc1* Het mice. NeuN-positive neurons in the white matter including the cingulum (cg) and external capsule (ec) were identified **(A)** and measured **(B,C)**. PV-positive white matter neurons **(D)** were also measured **(E,F)**. Scale bar = 100 μm in **(A,D)**. *N* = 8 WT and 7 Het mice. Results are means ± SEM. ***p < 0.001. cc, corpus callosum; LV, lateral ventricle; cg, cingulum; ec, external capsule.

Defects of neuronal migration might be manifested as altered white matter neuron density (Akbarian et al., 1996), we then examined the neurons in the white matter of Het mice (Figure 2). NeuN-positive neurons were evident in the cingulum and external capsule (Figure 2A). The density of NeuN-positive neurons was comparable between genotypes in the cingulum (Figure 2B); whereas in the external capsule, the density of NeuN-positive neurons was significantly greater in Het mice (Figure 2C). PV-positive neurons were sparsely distributed in the cingulum and external capsule (Figure 2D) and the densities of PV-positive neurons in WT and Het mice were similar in both areas (Figures 2E,F). Abnormalities in white matter neurons have been linked to mental disorders such as schizophrenia (Duchatel et al., 2016, 2019; Serati et al., 2019; Kubo, 2020), these results demonstrated, for the first time, an increased density of white matter neurons in a mouse model of schizophrenia.

### Expression of Subplate-Specific Markers in Cortical Layer VIb

**Figure 3:**
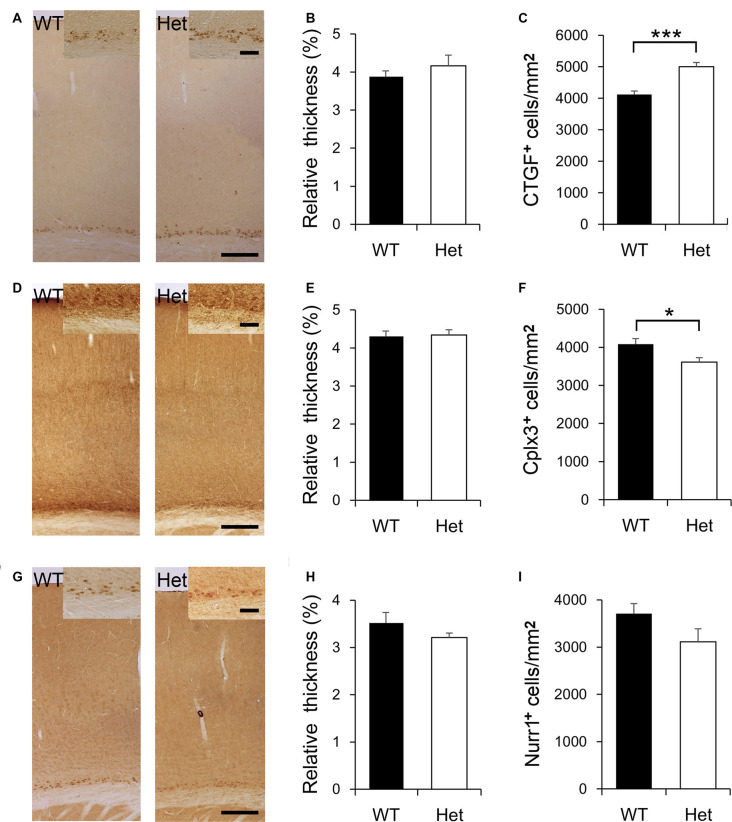
Expression of subplate-specific markers in cortical layer VIb. CTGF is expressed by the subplate neurons in cortical layer VIb of both WT and Het mice **(A)**. The relative thickness and density of CTGF-positive cells were measured and compared between genotypes **(B,C)**. Cplx3 is expressed by the subplate neurons in layer VIb **(D)**. The relative thickness of Cplx3-expressing cells was determined **(E)**; the density of these cells was measured and compared **(F)**. Nurr1 is evident in the nuclei of subplate neurons in layer VIb **(G)**. The relative thickness of Nurr1-positive signals was determined **(H)**; the density of these cells was measured and compared **(I)**. Scale bar = 200 μm in panels **(A,D,G)** and 50 μm in the inserts. *N* = 8 WT and 7 Het mice. Results are means ± SEM. *p < 0.05, ***p < 0.001.

During cortical development, most subplate neurons go through programmed cell death, and only a few neurons are left in the adult brain. Cortical layer VIb neurons are believed to be the remnant of subplate neurons in the adult rodent brain. There are several markers expressed in subplate neurons during the embryonic stage, and also expressed in adult layer VIb, such as connective tissue growth factor (CTGF), complexin-3 (Cplx3), and nuclear receptor-related 1 protein (Nurr1). It has been shown that these markers were not simultaneously expressed in the same subplate neuron population yet some overlapping still occurs (Mikhailova et al., 2017). In the present study, we picked these three markers to label subplate neurons in the adult brains (Figure 3), to examine if *Disc1* mutation affects the fate of subplate neurons. CTGF was expressed in cortical VIb of both WT and Het mice (Figure 3A), roughly composed 4% of the cortical thickness and this value was comparable between genotypes (Figure 3B). Notably, the density of CTGF-positive neurons in layer VIb was greater in the Het mice (Figure 3C). Cplx3 was also expressed in layer VIb (Figure 3D). Cplx3-positive zone composed about 4.2% of the cortical thickness in both genotypes (Figure 3E). The density of Cplx3-positive neurons in the layer VIb was lower in Het mice (Figure 3F). Nurr1 was expressed in the nuclei of some layer VIb cells (Figure 3G) and composed about 3.3% of the thickness of the cortex (Figure 3H). The relative thickness and density of Nurr1-positive neurons were similar between genotypes (Figures 3H,I). Here we reported the relative thickness of layer VIb was comparable between WT and Het mice; whereas the densities of some layer VIb neuron subtypes were altered in Het mice.

### Morphology of Layer VIb Neurons

**Figure 4:**
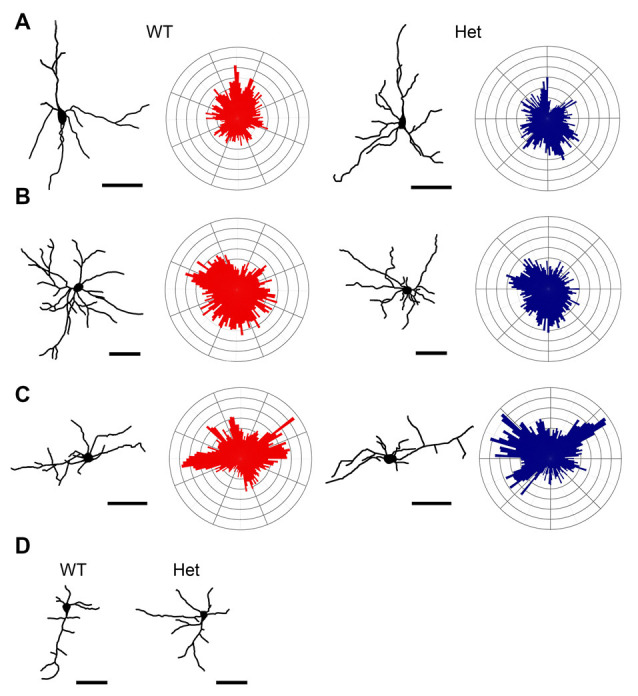
Classification of neurons in the layer VIb. Golgi-Cox impregnated layer VIb neurons were collected from WT and Het mice and reconstructed. Dendritic polar plots were also generated. Based on their morphological features, cells were classified as Type I pyramidal neurons **(A)**, Type II multipolar neurons **(B)**, Type III horizontal neurons **(C)**, and Type IV inverted neurons **(D)** of each type. Scale bar = 50 μm.

Subplate neurons have a large variety in cell morphology and a diversity in molecular marker expression (Kanold and Luhmann, 2010; Hoerder-Suabedissen and Molnár, 2013). In postnatal rodents, pyramidal-like, multipolar, horizontal, tangentially, and inverted subplate neurons have been discovered in the somatosensory cortex (Marx et al., 2017). In this study, Golgi-stained subplate neurons in layer VIb of the somatosensory cortex were collected from WT and Het mice and 3D reconstructed using Neurolucida software. The morphology of these neurons was classified based on quantitative analyses of their dendritic morphology. According to previously published reports (Marx and Feldmeyer, 2013; Marx et al., 2017), we classified the layer VIb neurons into pyramidal (Type I), multipolar (Type II), and horizontal (Type III) neurons. Few inverted (Type IV) subplate neurons were also identified. Different types of subplate neurons were demonstrated in Figure 4. In total, 72 and 77 subplate neurons were collected from WT and *Disc1* Het mice, respectively. The proportions of cell types were comparable between WT and Het groups (Table 1).

**Table 1:** Number and proportion of each type of layer VIb neurons.

	Type I pyramidal	Type II multipolar	Type III horizontal	Type IV inverted
WT mice	27 (37.5%)	26 (36.1%)	15 (20.8%)	4 (5.6%)
*Disc1* Het mice	29 (37.7%)	29 (37.7%)	17 (20.0%)	2 (2.6%)

*Neurons were collected from 5 WT and 5 Het mice*.

Parameters including soma size, primary dendrites, branch nodes, highest order, segment length, total dendritic length, and convex hull volume were measured, segment number and Sholl analyses (the numbers of intersections and branching nodes in relation to the distance from the soma) were conducted to evaluate the complexity of the dendritic trees.

### Type I Pyramidal Subplate Neurons

**Figure 5:**
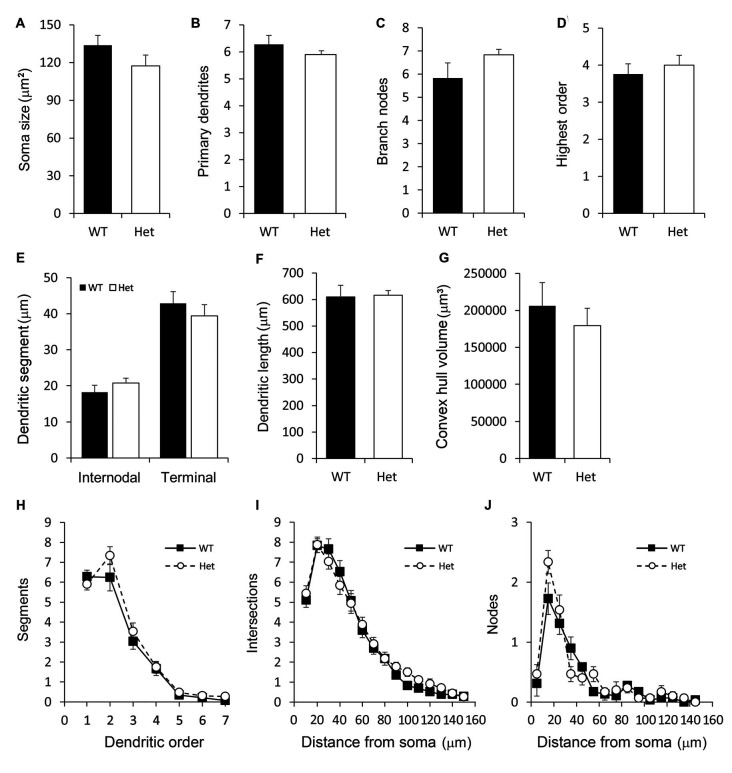
Morphometric analysis of Type I layer VIb neurons. Somatodendritic features of pyramidal layer VIb neurons including the soma size **(A)**, number of primary dendrites **(B)**, number of branch nodes **(C)**, highest order **(D)**, length of dendritic segments **(E)**, total dendritic length **(F)** and the convex hull volume **(G)** were measured. The complexity of the dendritic arbor was estimated by plotting the number of segments against the dendritic order **(H)** and using the concentric method of Sholl: the numbers of intersections **(I)** and nodes **(J)** were counted and plotted along the distance from the soma. Results are means ± SEM.

Pyramidal subplate neurons showed featured with the long and thick apical dendrite which branches several times towards the terminal and projects vertically to the cortical surface (Figure 4A). However, the actual length of the apical dendrite might be underestimated due to the limitation of the Golgi stain. A few short and thin basal dendrites emerged from the base of soma projected radially. The morphological features of the pyramidal subplate neurons in WT and Het groups were similar. No significant difference was noted between groups (Figure 5).

### Type II Multipolar Subplate Neurons

**Figure 6:**
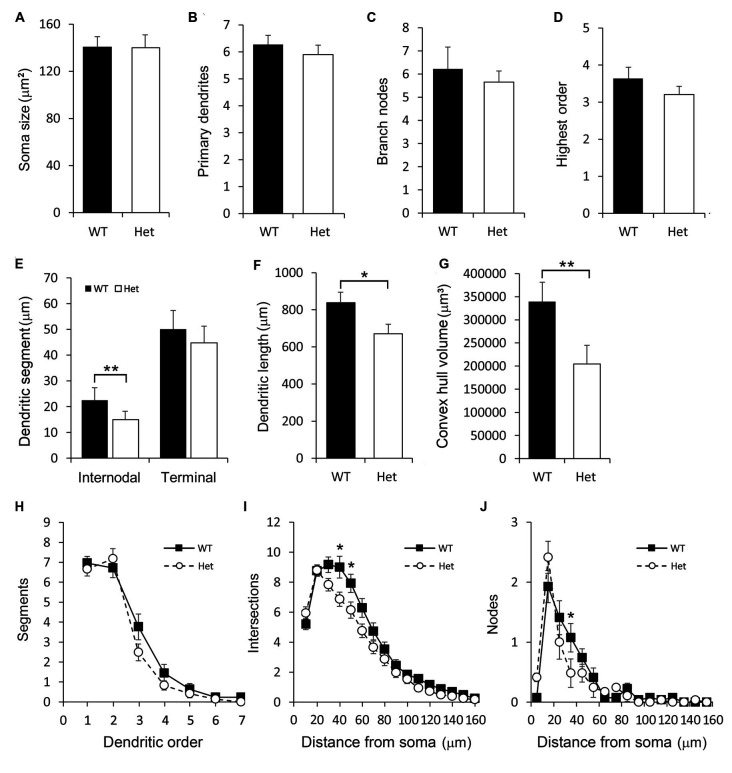
Morphometric analysis of Type II layer VIb neurons. Somatodendritic characteristics of multipolar layer VIb neurons including the soma size **(A)**, number of primary dendrites **(B)**, number of branch nodes **(C)**, highest order **(D)**, length of dendritic segments **(E)**, total dendritic length **(F)** and the convex hull volume **(G)** were measured. The number of segments was plotted against the dendritic order **(H)**. The numbers of intersections **(I)** and nodes **(J)** were counted and plotted along the distance from the soma. Results are means ± SEM. *p < 0.05, **p < 0.01.

Multipolar subplate neurons had multiple dendrites with varied orientations but no apical-like thick dendrite (Figure 4B). The soma size, numbers of primary dendrites and branch nodes as well as the highest order were not different between genotypes (Figures 6A–D). The length of internodal segments was significantly shorter in the *Disc1* Het group than in the WT group (Figure 6E), resulting in shorter total dendritic length (Figure 6F) and smaller convex hull volume in neurons collected from these mutant mice (Figure 6G). The numbers of segments were not changed in Het mice (Figure 6H). Sholl analysis showed reduced intersections and branch nodes in neurons of the *Disc1* Het group (Figures 6I,J), indicating reduced dendritic complexity and branching deficiencies in type II subplate neurons in Het mice.

### Type III Horizontal Subplate Neurons

**Figure 7:**
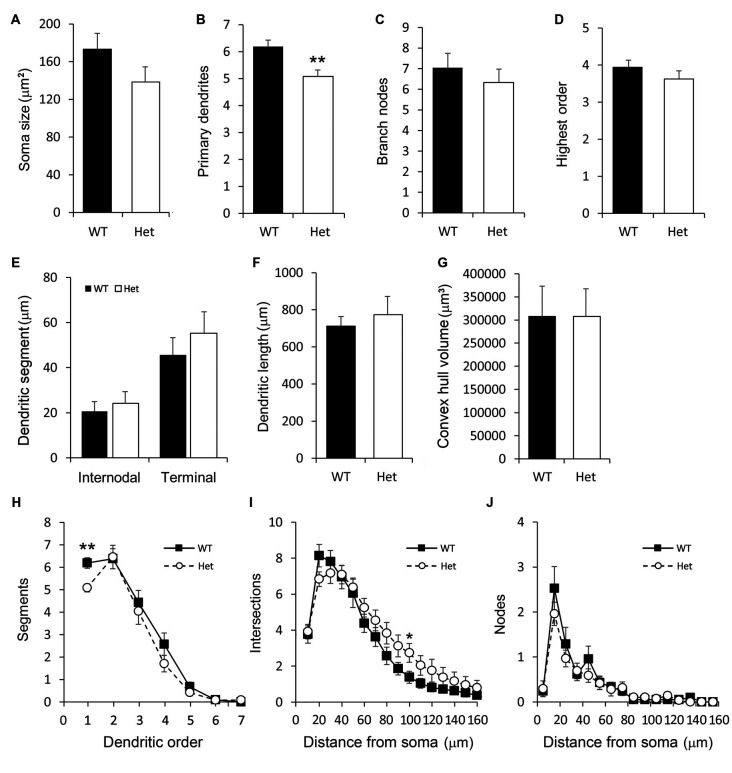
Morphometric analysis of Type III layer VIb neurons. Morphological features of horizontal layer VIb neurons including the soma size **(A)**, number of primary dendrites **(B)**, number of branch nodes **(C)**, highest order **(D)**, length of dendritic segments **(E)**, total dendritic length **(F)** and the convex hull volume **(G)** were measured. The numbers of segments **(H)**, intersections **(I)**, and nodes **(J)** were counted and plotted. Results are means ± SEM. *p < 0.05, **p < 0.01.

The neurons in this category had a thick apical-like main dendrite extending. However, the directions of the main dendrites were not towards the cortical surface but mainly towards the horizontal orientation, which paralleled the white matter beneath the cortex, and some oriented obliquely from the soma. The other dendrites had no particular orientations (Figure 4C). The number of primary orders was lower in the Het group compared to the WT control (Figures 7B,H), however, other morphological characteristics were comparable between groups (Figure 7).

### Type IV Inverted Subplate Neurons

Few neurons have an atypically oriented thick apical-like main dendrite extended from the base of soma and projected vertically towards the white matter (Figure 4D). Because of the rarity of this type of subplate neurons, we did not measure and compare these cells.

Together, our morphological study revealed a change in layer VIb subplate neurons, particularly the type II and type III subplate neurons, in Het mice.

## Discussion

Increased interstitial white matter neurons have been associated with psychiatric disorders including schizophrenia and autism (Duchatel et al., 2019; Serati et al., 2019; Kubo, 2020). The pathogenic role of aberrant subplate neuron remnants had been suggested (Judaš et al., 2010; Kostović et al., 2011). In the present study, we used a mouse model of schizophrenia, heterozygous *Disc1* mutant mice, to test this hypothesis. We observed increased white matter neurons, altered CTGF- and Cplx3-positive layer VIb neuron densities as well as reduced dendritic complexity in layer VIb neurons. Increased white matter neurons and aberrant subplate neuron remnants in *Disc1* mutants might contribute to altered brain functions in these mice.

Schizophrenia has been long recognized as dysfunctions in higher cognitive domains in humans. However, since the core symptoms include hallucination, defects in the sensory aspects might be relevant (Vukadinovic, 2012; Weilnhammer et al., 2020). Deficits in primary visual, auditory, and somatosensory cortices in schizophrenia patients have been reported (Fletcher and Frith, 2009; Huang et al., 2010, 2019; Molnár et al., 2014; Bordier et al., 2018; Daskalakis et al., 2020). We, therefore, in this study, decided to examine the properties of subplate neurons in the somatosensory cortex, which occupies a great cortical area with a substantial white matter thickness, in the rodent brain, for a proof of concept exploration.

### Increased White Matter Neurons in *Disc1* Het Mice

In the present study, we showed an increased density of NeuN-positive neurons in the white matter of *Disc1* Het mice, a haploinsufficiency model of schizophrenia (Juan et al., 2014; Baskaran et al., 2020). This finding was in line with previous reports of human studies: increased densities of white matter neurons in the postmortem brain tissues from patients with schizophrenia (Anderson et al., 1996; Kirkpatrick et al., 1999, 2003, 2006; Yang et al., 2011; Joshi et al., 2012; for review, see Duchatel et al., 2019; Serati et al., 2019; Kubo, 2020). We acknowledged that in these studies, the increased densities of white matter neurons were found in the dorsolateral prefrontal, orbitofrontal, parietal, anterior cingulate, and superior temporal regions. However, less attention had been paid to the primary sensory regions. Sensory processing defects were prominent in patients with schizophrenia (Javitt, 2009), cytoarchitectural elevations of the white matter in the sensory areas are encouraged. The density of white matter PV-positive neurons was very low and not changed in our model. However, in a rat model of schizophrenia, maternal immune activation paradigm, increased white matter somatostatin-positive inhibitory neurons have been noticed (Duchatel et al., 2016). Therefore, the role of inhibitory neurons in white matter is unneglectable (Joshi et al., 2012).

Altered white matter neurons might be resulted from impaired neuronal migration or programmed cell death during brain development (Akbarian et al., 1996; Kubo, 2020). Defects of neuronal migration are linked with brain disorders and Disc1 has been suggested to play a role in this process (Tomita et al., 2011; Narayan et al., 2013; Muraki and Tanigaki, 2015). In the present study, the increased NeuN-positive white matter neurons in adult *Disc1* mutant mice might reflect a Disc1-mediated defect in cortical neuron migration. In our model, there is a 25-bp deletion in exon 6 of the *Disc1* gene (Juan et al., 2014) which leads to a frameshift in the rest sequences including a great portion of the tail domain. LIS1 and NDEL1, components of the dynein complex that are associated with the DISC1 tail domain, are involved in the dynamics of the cytoskeleton and neuronal migration (Kamiya et al., 2005). However, in our *Disc1* Het mice, the impact size was minimal, not as significant as other *Disc1* knockdown models (Kamiya et al., 2005; Kubo et al., 2010; Steinecke et al., 2014). Compensatory responses might be activated to ensure proper cortical development in our and other *Disc1* mutant mice (Koike et al., 2006; Kuroda et al., 2011). Nevertheless, the defects may still occur in the white matter, during an earlier period.

White matter neurons receive glutamatergic thalamic inputs and could be modulated by orexin, neurotensin, and dopamine, suggesting a role of white matter neurons in sleep/arousal state control (Case et al., 2017). Increased density of white matter neurons might thus be relevant to dysregulation of sleep. Sleep/circadian rhythm disturbances have been considered as environmental risk factors related to various psychiatric disorders (Wulff et al., 2010; Jagannath et al., 2013). Such disturbances might interact with genetic risk factors and contribute to the manifestation of psychiatric disorders.

### Changes in Different Subsets of Layer VIb Subplate Neurons in *Disc1* Het Mice

Most subplate neurons go through programmed cell death with only 10–20% of neurons remaining in adulthood (Torres-Reveron and Friedlander, 2007; Luhmann et al., 2018). In rodents, most remaining subplate cells compose cortical layer VIb (Marx et al., 2017) and still receive both excitatory and inhibitory inputs and project to different cortex layers and thalamus (Torres-Reveron and Friedlander, 2007; Liao and Lee, 2012; Marx and Feldmeyer, 2013; Viswanathan et al., 2017; Hoerder-Suabedissen et al., 2018; Zolnik et al., 2020). The relative thickness of layer VIb in the cerebral cortex was comparable between genotypes; however, the densities of some subset subplate neurons were differentially altered in *Disc1* Het mice.

Subplate neurons that express CTGF are non-pyramidal layer VIb neurons (Zolnik et al., 2020) and the density of this subset was found to increase in Het mice. CTGF is a secreted extracellular matrix-associated protein that plays various roles in regulating cellular functions (Malik et al., 2015; Ramazani et al., 2018). Previous studies indicate an inhibitory role of CTGF in regulating the differentiation of oligodendrocytes (Stritt et al., 2009; Ercan et al., 2017). We showed an increased density of oligodendrocytes in the external capsule of forebrain-specific CTGF knockout mice (Yu et al., 2019), demonstrating a paracrine function of subplate neuron-derived CTGF. In this regard, increased CTGF-positive neurons in layer VIb might influence the maturation and function of oligodendrocytes in adjacent white matter. Pathological features in the white matter have been noticed in patients with schizophrenia (Chen et al., 2018; Raabe et al., 2019), and the role of DISC1 has been addressed (Miyata et al., 2015; Vasistha et al., 2019). Here, we provided evidence that supernumerary CTGF-positive subplate neurons in *Disc1* mutant mice might be a pathogenic sign leading to white matter dysfunction. However, the causal relationship between *Disc1* haploinsufficiency and CTGF expression in the layer VIb is still unknown.

Another subset of subplate neurons express Cplx3 (Hoerder-Suabedissen et al., 2013) that project to the septal region of layer IV and medial posterior thalamic nucleus. These Cplx3-expressing subplate neurons are thus positioned to integrate thalamocortical and corticothalamic circuits (Viswanathan et al., 2017). In this point of view, the reduction of Cplx3-positive layer VIb neurons might affect the processing of sensory information in *Disc1* Het mice which might model the sensory processing deficiency in patients with schizophrenia (Javitt, 2009).

The mechanisms underlying the differential changes among the subsets of layer VIb neurons in Het mice are not known. Since we only quantified the densities of layer VIb neurons at a single time point, the developmental trajectories of different subset neurons are not clear. Since most subplate neurons are removed during cortical development, the differential changes among CTGF-, Cplx3- and Nurr1-positive cells might be attributed to cell-type-specific survival mechanisms (Pfisterer and Khodosevich, 2017). We should examine the early signs of programmed cell death (Yamaguchi and Miura, 2015) at different time points in a subset-specific manner. The role of Disc1 in these processes still awaits to be explored.

### Morphological Changes in Subplate Neurons of *Disc1* Het Mice

Type I pyramidal subplate neurons, like the pyramidal neurons in layer VIa in morphology and function, have corticothalamic and corticocortical projections (Thomson, 2010; Marx and Feldmeyer, 2013). A recent study revealed that pyramidal layer VIb subplate neurons are Drd1a-positive and project to layers I and V and high order thalamus (Zolnik et al., 2020). The dendritic morphology of Type I subplate neurons was similar between WT and Het mice, implying that the function of these neurons might be spared in the mutants. However, due to the limitation of Gogi-stain and sample collection, the morphometric features of the apical dendrites might be underestimated. Besides, the detailed dendritic features such as dendritic diameter, density, and shape of dendritic spines, and ultrastructural characters have not been tested. We are not able to conclude this notion.

The multipolar and horizontal (Types II and III) subplate neurons are non-pyramidal layer VIb neurons that receive inputs from various cortical and subcortical sources (Zolnik et al., 2020). Some of these cells, especially the Type II cells, might be CTGF-positive layer VIb neurons. The reduced dendritic field in these cells explained the increased density of CTGF-positive neurons in mutant mice while the thickness of the CTGF-positive layer was comparable to that in WT mice. The dendritic length, complexity, and volume of the convex hull in Type II multipolar subplate neurons and the number of primary dendrites in Type III horizontal neurons were reduced in Het mice, indicating a reduced receptive field of synaptic transmission between layer VIb neurons and other cortical/subcortical neurons in *Disc1* mutants. Future studies involving the projections of neurons and synaptic connectivity are required to clarify the functional roles of subplate neurons in different subpopulations. Subplate neurons in younger mice should be identified to advance our knowledge of Disc1-mediated dendritic growth or pruning. Further, white matter neurons can be classified and compared with layer VIb neurons.

### Patterns of Cortical Neurons

In the somatosensory cortex, the densities and distributions of NeuN- and PV-positive neurons were similar between WT and Het mice, except for a subtle increase in the proportion of NeuN-positive neurons in bin 8, the cortical layer IV, of *Disc1* Het mice. Layer IV neurons are the major recipients of thalamic and cortical inputs and play a critical role in sensory processing. Altered neuronal distribution in layer IV together with the changes of layer VIb subplate neurons might suggest a defect in sensory function. We should elaborate on this possibility using tactile-related behavioral tests (Lee, 2009; Arakawa et al., 2014) in *Disc1* Het mice.

## Conclusion

Subplate neurons regulate the radial migration of excitatory neurons in the cerebral cortex (Ohtaka-Maruyama et al., 2018) and modulate the thalamocortical and corticothalamic circuits that have been associated with cognitive functions. The subplate-specific gene expression profile is related to mental disorders including autism and schizophrenia (Hoerder-Suabedissen et al., 2013). Our results showed *Disc1* haploinsufficiency-mediated pathogenic features in the white matter and layer VIb that can be associated with schizophrenia. This mouse model should be used to explore the developmental trajectory to replicate the at-risk subjects of developmental neuropsychiatric disorders and develop a preventive strategy. Our pilot study should also encourage the exploration of subplate-specific gene and network study during cortical development.

## Data Availability Statement

The raw data supporting the conclusions of this article will be made available by the authors, without undue reservation.

## Ethics Statement

The animal study was reviewed and approved by Institutional Animal Care and Use Committee of the College of Medicine, National Taiwan University.

## Author Contributions

S-HT and C-YT conducted the experiments. S-HT analyzed the data and composed the draft of the manuscript. L-JL finalized the manuscript. All authors contributed to the article and approved the submitted version.

## Conflict of Interest

The authors declare that the research was conducted in the absence of any commercial or financial relationships that could be construed as a potential conflict of interest.
